# Pyorrhea Alveolaris; Its Prevention and Cure

**Published:** 1909-05-15

**Authors:** Joseph W. Wassall

**Affiliations:** Chicago


### PYORRHEA ALVEOLARIS; ITS PREVENTION AND CURE.

BY JOSEPH W. WASSALL, M.D.,D.D.S., CHICAGO

Very properly described in the annual address of our out-going president at the last meeting as the "Scourge of Human Mouths," pyorrhea alveolaris is in its destructive potency the peer of dental caries. Susceptibility to both these maladies is governed by the subject's age, for, generally speaking, caries is a disease of youth, while the attacks of alveolitis occur at maturity.

The pathology of pyorrhea alveolaris is but imperfectly understood, and its etiology is still a mystery, notwithstanding the opportunities afforded for its observation and study by its almost universal prevalence. Investigators differ widely as to whether it is a local or systemic affection or whether it is a combination of both, or whether there are not the two distinct types of the malady, local and systemic. Adherents of the various schools of belief as to its nature and etiology have written voluminously, and it must be confessed convincingly to establish their varying speculations, and to upset the tenets of opposing doctrinaire.

Until the essential nature and real causes of this many-sided affection are known, the ordinary practitioner is left to work out his own salvation, availing himself of whatsoever of the teaching of others appeals to his best judgment, combining with it the results of his own study and clinical experience. It follows, therefore, that in the present state of knowledge his treatment must be empirical—as, in fact, it is.

There is a widely prevalent and deeply rooted notion that excrementitious matter circulating in the blood, due to malnutrition, suboxidization of metabolism, or faulty innervation is the paramount cause. Pierce, Kirk and more recently Endelman are strong believers in this theory. But all of us have seen patients with pyorrhea entirely and permanently cured by local treatment alone. Kirk and Endelman ingeniously attribute the formation of uratic deposits upon the roots of teeth to a localized lessened alkalinity of tissue at the point where the concretion occurs; but the arguments set forth that motion in a part lessens alkalinity is far-fetched and hardly applicable to firmly socketed teeth. It is a far cry from the red muscular fibers of the great toe to the parsimonious distribution of white fibers found in the peridental membrane. The every-day observor of cases is impressed by the great fact of susceptibility and immunity. Upon what do these conditions depend? That predisposition to this affection in the organs is the controlling factor is borne in upon us every day. But in what this predisposition consists we are in the dark. We know that life-long sufferers from arthritism are entirely free from pyorrhea alveolaris, while subjects in robust, perfect physical health are its easy victims. That a depraved physical condition encourages any local inflammation cannot be disputed. That it must be corrected is obvious, but internal treatment alone will not suffice to cure any of them, let alone pyorrhea alveolaris. It is generally accepted that pyorrhea alveolaris in common with other modern diseases is one of the penalties exacted by civilization. It is the direct result of a departure from the savage state of man, when his dental apparatus performed its full physiological functions. It is a well-known biological fact that the teeth of man or beast will not remain solidly fixed in their osseous and gingival seats unless they receive the hard usage nature ordained, or its artificial equivalent. This deficiency must be supplied if we would have the dental organism survive.

The equivalent of the strenuous use of the teeth and jaws which primitive man was perforce obliged to employ to maintain life is a return to harder food stuffs and the application of artificial friction.

To establish a new regimen in the lives of our patients is a task from which the bravest might shrink. It is a herculean undertaking to reform dietetics in these days, when the luxury and ease of good living invite our indulgence on every side.

The advocacy of a regimen of plain hard food is an old propaganda, but it needs to be promulgated with all the force and authority of a new cult, championed by a masterful leader, who will be supported by all the authority of the dental and medical world. But such a drastic reform in the habits of life being not immediately practicable, or being too Utopian, we must rely upon the other factor in prophylaxis; namely, artificial friction. The best means by which this is applied now at our command seems to be stiff-bristled brushes. The entire gingival surfaces of the alveolus are to receive the rubbing —the motion of the brush to be vertically downward on the maxilla, and upward in the mandible. A three-minute sand-glass (egg boiler) should be used to indicate the duration of brushing. The full time as stated should be employed without shirking—twice daily. A conscientious practice of the above recommendation, it is the belief of the author, would practically abolish the disease under consideration. But these ideal conditions do not obtain, hence we are brought to the last part of my paper; namely, the cure of pyorrhea alveolaris.

First, there are so many cases so far advanced that they should never be treated—the teeth should be extracted. By error of judgment or too sanguine expectations, efforts to prolong the existence of teeth seriously affected only afford temporary relief, and by a recrudescence of odd conditions open the way to a spread of infection.

When the disease has advanced far upon one member, the exposed uneven surfaces of the root inevitably harbor growths of pathogenic microorganisms. It is, therefore, futile and unwarranted to make efforts to prolong their existence. This is as true of incisors as of molars.

In these days of really reliable bridging to supply lost teeth, it is no longer a calamity to sacrifice one or two members of a dental arch to protect the whole.

The clearing of the mouth of condemned offenders having been accomplished, measures to restore normal conditions in other affected members of the arch are in order.

The treatment consists of surgery, medication, splinting, and instructions in personal hygiene to prevent recurrence. As a preliminary procedure, the mouth and pockets are thoroughly sprayed under high pressure. Surgery involves correcting occlusion—always diminishing the excess which the disease usually has produced, and relieving the liability to more than very slight contact with opposing teeth. This at once removes a source of irritation, and is in accord with the general principle that all inflammations subside more easily in physiological rest. The removal of the concretions is then accomplished.

Medication is not, to the writer's mind, an important matter. Measures to remove all foreign matter, both infective and mechanical, should, of course, be taken. This, it is needless to say, is imperative. Pyrogenic membrane lining pockets or sacks should be curetted and cauterized in the first treatment, but this should not be repeated.

The case is now ready for immobilization by splinting, if the conditions require it. This is an important aid, but the comfort thereby afforded should not lull the operator into false security, tempting him to conserve unworthy teeth, as is so frequently the case.

The patient should be instructed as to the curative value of brush friction on the gums, and absolute cleanliness. Until the pockets are healed, an automizer should be used three times daily, at home. Prolonged brush friction with cold salt water, at least three minutes twice daily, will keep the pockets evacuated and prevent the formation of bacterial colonies at the gingival margin or in the unhealed pockets. Demonstrate how the brush is to be applied directly to the whole surface of the alveolus and the importance of brush movements only from apical ends to necks.

The results of this extreme prolonged brushing with hard brushes and cold water have been so surpassingly successful in curing and preventing recrudescence of pyorrhea alveolaris that I am convinced that it has a more profound influence than mere asepsis and toughening of the soft tissues, which was the object first sought. It is fair to presume that even the most dexterous and expert manipulator of the scaler of tooth roots will frequently fail to detect and remove deposits entirely. The inference is, therefore, that as these cases get well the deposits are absorbed. It is my belief and theoretical conviction that the extreme brushing recommended happily stimulates the activities of the cementoclasts in the peridental membrane to an extent which has heretofore been unsuspected.—*Dental Review.*

